# Editorial: Healthy aging: The role of disease burden and functional ability

**DOI:** 10.3389/fmed.2023.1150877

**Published:** 2023-02-14

**Authors:** Demosthenes Panagiotakos, Victor Gkotzamanis, Josep Maria Haro, Stefanos Tyrovolas

**Affiliations:** ^1^School of Health Sciences and Education, Harokopio University, Athens, Greece; ^2^Research and Development Unit, Parc Sanitari Sant Joan de Déu, Sant Boi de Llobregat, Barcelona, Spain; ^3^Centro de Investigación Biomédica en Red de Salud Mental (CIBERSAM), Instituto de Salud Carlos III, Madrid, Spain; ^4^Facultat de Medicina y Ciencias de la Salut, Universitat de Barcelona, Barcelona, Spain; ^5^WHO Collaborating Centre for Community Health Services (WHOCC), School of Nursing, The Hong Kong Polytechnic University, Kowloon, Hong Kong SAR, China

**Keywords:** healthy aging, burden of disease, functionality, comorbidities, older adults

The aging population of the world is rapidly increasing nowadays. By 2050, those over 65 are expected to make up 17% of the global population. This demographic shift, common in most countries, is caused by both a rise in life expectancy and a decrease in birth rate. At a biological level, aging is linked to the gradual accumulation of damage in cells and molecules (1). Over time, this damage can result in a decline in normal functioning, an increased susceptibility to disease, and a general decline in human function, leading to dependence and ultimately death. However, the aging process varies greatly among individuals, depending on factors such as lifestyle, exposure to risk, genetics, socioeconomic background, and many others, both known and unknown. In other words, some people may maintain good physical and mental health as they age, while others of the same age may require assistance with basic needs. Given that this is a widespread global issue, strategies to promote healthy aging are a significant challenge for the scientific community. To this end, it is crucial to gain a clear understanding of the concept of healthy aging in order to identify its determinants and implement policies to promote it.

## Defining healthy aging

The concept of healthy aging has been defined in various ways over the years. These definitions range from the simple idea of the absence of disease and disability to more complex concepts that include subjective satisfaction (2), emotional wellbeing (3), and maintaining social participation and functionality (4). Despite the differences in the various approaches to healthy aging and the importance of each factor, it is widely accepted that it is a complex concept that should encompass more than just physical and mental health. The most recent definition by the World Health Organization (WHO) in 2015 defines healthy aging as “the process of developing and maintaining the functional ability that enables well-being in older age” (5). This includes the ability to meet basic needs independently, maintain social relationships, and contribute to society. This definition emphasizes the importance of functional capacity and goes further by identifying the factors that make up that capacity, including the intrinsic capacity of the individual, the characteristics of the environment they live in, and the interaction between the two. The intrinsic capacity includes all the mental and physical abilities of the individual that allow them to perform daily activities and is greatly influenced by the presence of disease and disability. The environment includes factors such as housing conditions, transportation, facilities, and social services, as well as relationships and social and health policies. These two factors are closely related as the environment can compensate for the decline of intrinsic capacity that comes with age, while differences in intrinsic capacity among individuals may be partly attributed to the cumulative effect of the environment they live in. Therefore, research on healthy aging must take into account both intrinsic capacity and the environment as it is their interaction that ultimately defines the functional ability of the individual.

## Disease burden vs. functional ability

Despite progress in defining healthy aging, researchers often rely on biomarkers and the accumulation of co-morbidities to develop metrics for healthy aging (6, 7). These metrics can be useful tools for research in the field, as the intrinsic capacity is related to the presence of disease and disability. However, they fail to capture the entirety of the concept of healthy aging as defined by the WHO, as many chronic conditions, when well-controlled, may have little impact on the wellbeing of the individual, and they do not take into account the role of the environment. On the other hand, some researchers have developed metrics that represent the functional capacity of the individuals. In the context of the Aging Trajectories of Health: Longitudinal Opportunities and Synergies (ATHLOS) project, the researchers developed a metric of healthy aging, based on information from questionnaires assessing the functional capacity of the participants using factor analysis and machine learning techniques (8). Similarly, in the context of the Hellenic Longitudinal Investigation of Aging and Diet (HELIAD) the researchers developed a metric of functional ability using an Item Response Theory (IRT) approach (9). Although such metrics require the use of more sophisticated techniques, they have some inherent advantages compared to other health metrics and biomarkers in the research of healthy aging. Firstly, they are, by definition, measures of functional capacity, including an assessment not only of the intrinsic capacity but also its interplay with the environment, which is the core of the WHO definition of healthy aging. Additionally, with this approach, researchers may investigate the impact of disease burden in healthy aging which would not be possible if the presence of a disease or a biomarker that is associated with it was part of the metric itself. As a result, such metrics can be used in complex models that will include socioeconomic, biological and clinical factors in order to identify potential determinants of healthy aging (10–13).

## Conclusion

Healthy aging is an elusive concept and various definitions have been proposed through the years. The WHO emphasized the functional capacity of individuals as a key aspect of healthy aging in its 2015 definition. This functional capacity is determined by both the individual's intrinsic abilities and the environment they live in. It is important to note that disease burden alone is not a comprehensive indicator of healthy aging and can be misleading. Therefore, research on healthy aging should focus on functional ability in order to identify factors that can promote a trajectory of healthy aging.

## Author contributions

Conceptualization, writing—original draft preparation, and writing—review and editing: DP, VG, JH, and ST. Supervision: DP. All authors contributed to the article and approved the submitted version.
